# Integrated taxonomy: traditional approach and DNA barcoding for the identification of filarioid worms and related parasites (Nematoda)

**DOI:** 10.1186/1742-9994-6-1

**Published:** 2009-01-07

**Authors:** Emanuele Ferri, Michela Barbuto, Odile Bain, Andrea Galimberti, Shigehiko Uni, Ricardo Guerrero, Hubert Ferté, Claudio Bandi, Coralie Martin, Maurizio Casiraghi

**Affiliations:** 1 Dipartimento di Patologia Animale, Igiene e Sanità Pubblica Veterinaria, Sezione di Patologia Animale e Parassitologia, Università degli Studi di Milano, via Celoria 10, 20133 Milano, Italy; 2 Dipartimento di Biotecnologie e Bioscienze, ZooPlantLab, Università degli Studi di Milano Bicocca, P.zza della Scienza 2, 20126 Milano, Italy; 3 Parasitologie Comparée et Modèles expérimentaux USM 307, Muséum National d'Histoire Naturelle, 75231 Paris Cedex 05, France; 4 Department of Medical Zoology, Osaka City University Medical School, 1-4-3 Asahimachi, Abeno-ku, Osaka 545-8585 Japan; 5 Instituto de Zoologia Tropical, Faculdad de Ciencias, Universidad Central de Venezuela, PO Box 47058, 1041° Caracas, Venezuela; 6 JE 2533 USC AFSSA «Vecpar» UFR de Pharmacie, 51 rue Cognacq-Jay, 51096 Reims, France

## Abstract

**Background:**

We compared here the suitability and efficacy of traditional morphological approach and DNA barcoding to distinguish filarioid nematodes species (Nematoda, Spirurida). A reliable and rapid taxonomic identification of these parasites is the basis for a correct diagnosis of important and widespread parasitic diseases. The performance of DNA barcoding with different parameters was compared measuring the strength of correlation between morphological and molecular identification approaches. Molecular distance estimation was performed with two different mitochondrial markers (*coxI *and 12S rDNA) and different combinations of data handling were compared in order to provide a stronger tool for easy identification of filarioid worms.

**Results:**

DNA barcoding and morphology based identification of filarioid nematodes revealed high coherence. Despite both *coxI *and 12S rDNA allow to reach high-quality performances, only *coxI *revealed to be manageable. Both alignment algorithm, gaps treatment, and the criteria used to define the threshold value were found to affect the performance of DNA barcoding with 12S rDNA marker. Using *coxI *and a defined level of nucleotide divergence to delimit species boundaries, DNA barcoding can also be used to infer potential new species.

**Conclusion:**

An integrated approach allows to reach a higher discrimination power. The results clearly show where DNA-based and morphological identifications are consistent, and where they are not. The coherence between DNA-based and morphological identification for almost all the species examined in our work is very strong. We propose DNA barcoding as a reliable, consistent, and democratic tool for species discrimination in routine identification of parasitic nematodes.

## Background

The identification of living species is one of the major goals of modern biology. Species can be delimited only in relation to other species, it is trivial, but many discriminators and species concepts can be used for this purpose. Molecular data have become widely used to aid rapid assessment of species diversity, and the DNA barcoding initiative [1] is one prominent line of research within this field, coordinated by the Consortium for the Barcode of Life (CBoL, ). DNA barcoding involves rapid sequencing of one or a few genes from several representatives of a species, as well as comparisons of these sequences within and between species. The method has revealed examples of cryptic species diversity in various taxa [2,3]. DNA barcoding aims at the development of a universal, standardized and economical tool, but the fear is that to gain a sufficient accuracy the sampling should be massive, and, accordingly, the PCR and sequencing efforts expensive. Finding a balance between standardization, low costs and accuracy is difficult, and researchers have to take key decisions on the level of accuracy they want to get and the costs they can reasonably sustain.

A major strength of DNA barcoding is that it allows correlating any life stages of a living organism, or also a small part of it, to a single molecular entity (Molecular Operational Taxonomic Unit, MOTU; *sensu *Floyd *et al*. [4] and does not necessarily require taxonomy skilled personnel, at least in the step of the molecular data generation, to be used in the analysis. Nevertheless, the power and pitfalls of the DNA barcoding approach have not yet been fully evaluated. In particular, the proper methods to analyse DNA barcoding data are still under study (e.g. see the progress at CBoL working groups, ).

*coxI *sequences are widely used for DNA barcoding of metazoans, but several markers have been proposed as putative barcodes [5,6] and different authors underline the importance of a DNA barcoding approach based on multiple markers [7,8]. Ribosomal mitochondrial genes are often used as alternatives to *coxI *marker for different reasons: easy to amplify, good source of synapomorphies in loop regions and abundance of sequences in databases [8-10].

Predicted problems in DNA barcoding studies include: poor taxonomy (e.g. single species misidentified as two or more species and *viceversa*); insufficient sampling within a taxon, or insufficient sampling of taxa (see for instance [11] and consequent criticisms in [12-14]); polyphyletic or paraphyletic species [15].

This work focuses on an integrated approach at the identification of a group of nematodes, belonging to the order Spirurida, which includes the relevant superfamily Filarioidea. Several species of filarioid nematodes are agents of tropical diseases both for human and other animals of economical value. All the filarioids are transmitted through haematophagous vectors in which they span different juvenile stages [16-18]. The identification of these juvenile stages is a necessary condition for establishing the potential of transmission in endemic areas but it is difficult, due to the small size of the juvenile stages (about 1 mm) and paucity of characters. Identification of juvenile stages is also useful to detect any possible emergent zoonotic filarial disease at its beginning. Laboratories typically deals with fragments of parasitic nematodes recovered from host tissues, or with specimens representing a single developmental stage, and the diagnostic characters are often not present in these pieces of worms.

The identification of filarioid and related nematodes via DNA barcoding is an ambitious and desirable goal for many reasons: 1) a fast identification engine, available not only for taxonomists, but validated by them, is useful for quicker diagnoses of filariasis; 2) filarioids cause diseases of high relevance in medical and veterinary fields throughout the world; 3) DNA barcoding can be useful for those cases of difficult or impossible identification by traditional procedures, such as co-infections with more than one filarioid species (e.g. *Onchocerca volvulus *and *Loa loa*; see [19]); 4) parasites conferred to diagnostic laboratories are often of poor quality due to the difficult of sampling adults and undamaged organisms; 5) the model of filarioid nematodes being based on a very good classical taxonomy (starting from [20]) allow to avoid (as much as possible) problems of 'bad taxonomy' (see discussion in [15]); 6) DNA barcoding can offer a reliable method for the identification of filarioid nematodes in vectors, allowing widespread campaigns of epidemiological surveys; 7) nematode biodiversity is still highly underestimated both at the morphological and molecular level [21], and a molecular approach will speed up the estimation of this taxonomic diversity [3].

Despite molecular data from representatives of filarioids and deposited in public databases are quite abundant for species of medical or veterinary relevance, very few DNA barcodes are available if compared with other taxa of similar dimensions; this is mostly caused by the difficulties of sampling many species of parasitic nematodes. Most of these DNA sequences are relative to mitochondrial genes, in particular 12S rDNA and *coxI *[9,22].

Here we present a double approach (morphological and molecular) to the taxonomic identification of filarioids and related nematodes on the widest (in term of species number) molecular collection of these parasites ever achieved. Morphological identification was performed by well known international experts, whilst molecular distance estimation was performed with two different mitochondrial markers (*coxI *and 12S rDNA) and under different combinations of data handling (see below). The performance of DNA barcoding with different parameters was compared measuring the strength of correlation between morphological and molecular identification approaches. In order to provide a useful tool for easy identification of filarioid nematodes this work aims to answer the following questions: 1) which is the performance of DNA barcoding on filarioids and related nematodes? 2) which is the better marker (between *coxI *and 12S rDNA) for identification these organisms at the species level? 3) can DNA barcoding be a useful tool for detection of putative new species?

## Methods

### Biological samples, DNA extractions, PCR conditions, DNA sequencing and accession numbers

Filarioids and related nematodes belong to the order Spirurida, a group of heteroxenic parasites with arthropod intermediate hosts [20,23]. In vertebrate definitive hosts, they are found in the digestive tract or in other different tissues, from the lymphatic to blood vessels and heart chambers, from abdominal and thoracic cavities to skin and subcutaneous tissues. Well preserved biological samples are not easily obtained for these parasites, since dissection of vertebrate hosts is generally required for collection. We emphasize that most of the specimens for which we generated DNA sequences derive from wild naturally infected hosts, and most of the samples have been recovered at necropsy. In spite of these difficulties, we have included in this study the most important filarioid parasites of humans and other animals, including *Onchocerca volvulus*, agent of human river blindness, *Wuchereria bancrofti *and *Brugia malayi*, agents of human tropical elephantiasis, *Loa loa*, agent of human ocular filariasis, *Dirofilaria immitis*, agent of heartworm disease of dogs and cats, plus a collection of specimens recovered from the tissues of wild animals such as bats, ungulates, monkeys, tropical toads, reptiles and birds, collected all around the world (see Table 1 for a summary of the species considered in this study; for further details on these organisms see additional file 1: 'Investigated specimens').

**Table 1:** List of the species included in this study. List of nematodes species included in this study and their relevance in human (H), veterinary (V) or zoonotic (Z) parasitic diseases. Species used as models in researches are also indicated (model).

**Species**	**Relevance**
*Acanthocheilonema reconditum *(Grassi, 1890)	V
*Acanthocheilonema viteae *(Krepkogorskaya, 1933)	model
*Brugia malayi *(Brug, 1927)	H
*Brugia pahangi *(Buckley & Edeson, 1956)	V, model
*Cercopithifilaria bulboidea *Uni & Bain, 2001	
*Cercopithifilaria crassa *Uni, Bain & Takaoka, 2002	
*Cercopithifilaria japonica *(Uni, 1983)	
*Cercopithifilaria longa *Uni, Bain & Takaoka, 2002	
*Cercopithifilaria minuta *Uni & Bain 2001	
*Cercopithifilaria multicauda *Uni & Bain, 2001	
*Cercopithifilaria roussilhoni *Bain, Petit & Chabaud, 1986	
*Cercopithifilaria shohoi *Uni, Suzuki & Katsumi, 1998	
*Cercopithifilaria tumidicervicata *Uni & Bain, 2001	
*Dipetalonema gracile *(Rudolphi, 1809)	
*Dirofilaria (Dirofilaria) immitis *(Leidy, 1856)	V, Z, model
*Dirofilaria (Nochtiella) repens *Railliet & Henry, 1911	V, Z
*Filaria martis *Gmelin, 1790	
*Foleyella furcata *(Linstow, 1899)	
*Litomosa westi *(Gardner & Smith, 1986)	
*Litomosoides brasiliensis *Lins de Almeida, 1936	
*Litomosoides galizai *Bain, Petit, Diagne, 1989	
*Litomosoides hamletti *Sandground, 1934	
*Litomosoides scotti *Forrester & Kinsella, 1973	
*Litomosoides sigmodontis *Chandler, 1931	model
*Litomosoides yutajensis *Guerrero, Martin & Bain, 2003	
*Loa loa *(Cobbold, 1864)	H, model
*Loxodontofilaria caprini *Uni & Bain, 2006	
*Mansonella (Cutifilaria) perforata *Uni, Bain & Takaoka, 2004	
*Mansonella (Tetrapetalonema) atelensis amazonae *n. subsp. Bain & Guerrero, 2008	
*Ochoterenella *sp. sensu Casiraghi et al., 2004	
*Onchocerca dewittei japonica *Uni, Bain & Takaoka, 2001	Z
*Onchocerca eberhardi *Uni & Bain, 2007	
*Onchocerca gibsoni *(Cleland & Johnston, 1910)	V, model
*Onchocerca lupi *Rodonaja, 1967	V
*Onchocerca ochengi *Bwangamoi, 1969	V, model
*Onchocerca skrjabini *Ruklyadev, 1964	
*Onchocerca suzukii *Yagi, Bain & Shoho, 1994	
*Onchocerca volvulus *(Leuckart, 1893)	H
*Piratuba scaffi *Bain, 1974	
*Setaria digitata *(Linstow, 1906)	V, Z
*Setaria equina *(Abildgaard, 1789)	V
*Setaria labiatopapillosa *(Alessandrini, 1848)	V
*Setaria tundra *Issaitshikoff & Rajewskaya, 1928	
*Spirocerca lupi *(Rudolphi, 1809)	V
*Thelazia callipaeda *Railliet & Henry, 1910	V
*Thelazia gulosa *(Railliet & Henry, 1910)	V
*Thelazia lacrymalis *(Gurlt, 1831)	V
*Wuchereria bancrofti *(Cobbold, 1877)	H

All the biological material analysed have been stored following the procedures specified in the Biorepositories initiative  and belong to the collection identified as ':zpl' of MIB institution (which represents a confirmed record at Biorepositories initiative). Details on parasite species included in this work are given in additional file 1: 'Investigated specimens'

DNA extraction procedures, PCR conditions and sequencing of amplified DNA fragments were performed following standard procedures (details are found in the additional file 2: 'Experimental conditions'). Primers used for amplification are: *coxI*: coIintF and coIintR [22]; 12S rDNA: 12SF and 12SR [9]. The sequences generated have been deposited in the EMBL Data Library according to the EBI Barcoding Procedure (see details available at ) under the following accessions: *coxI*: [GenBank:AM749226–AM749298, GenBank:AM886173]; 12S rDNA: [GenBank:AM779769–AM779855]. The detailed list of accession numbers is found in the additional file 1: 'Investigated specimens'.

The datasets have been deposited in EMBL data alignment under the following accessions: *coxI *alignment [GenBank:ALIGN_001178]; 12S rDNA alignment: [GenBank:ALIGN_001179].

### The morphological identification procedure

For species identification, a morphological anatomical analysis is performed with worms cleared in lactophenol and using an optical microscope equipped with a *camera lucida*. The characters studied have been validated since years [24] and are the basis of the key of identification [20]. They include the measurements, the number and disposition of the sensory papillae on head and male tail, the different parts of the digestive tract and of the genital apparatus. A series of other characters have been introduced for precise identification; these are thought to be important during mating and able to discriminate close species: the cuticular ornamentation of male posterior region, or *area rugosa*, which acts as anti-slit system; the spicule distal extremities; the muscular-hypodermal anatomy. In filarioids, the first stage larva or microfilaria is a good discriminative character and is particularly studied: specimens are fixed in extension and measured; the cephalic hook and other cuticular head ornamentation are analysed as well as caudal extremity. For the correct observation of many characters manipulations are necessary: dissection of spicules and ovijector, head cut and orientation in front view, etc. (see [25-27]).

### Definition of molecular datasets

The DNA sequences used in this study were obtained by direct sequencing of PCR products or collected from GenBank; only sequences meeting a priory defined criteria of length, position, similarity and taxonomy were analysed. Each DNA sequence analysed belong to one of the four following groups: (1) sequences originated from organisms morphologically identified by international experts of our group; (2) sequences collected from GenBank and morphologically identified by international experts not affiliated to our group; (3) sequences originated from organisms collected by our group and morphologically undetermined; (4) sequences collected from GenBank and whose identification process is not certainly based on morphology.

DNA sequences were partitioned in three types of datasets (called here A, B and C) based on the analyses to be performed. In order to carry out DNA barcoding study with the standard marker *coxI *two datasets called A and B have been produced.

Dataset A encompasses only sequences derived from specimens for which morphological identification was sure (cases 1 and 2) and was used to test the coherence between morphological and molecular approaches following a 'classical' DNA barcoding analysis: generation of a Kimura 2-parameters (K2P; [28]) distance graph and cumulative error plots. *coxI *dataset A includes 151 sequences 627 bp long representing 46 morpho-species (with an average of 3.3 specimens per species; standard deviation 3.4; range: 1–20).

Dataset B encompasses all *coxI *sequences available (cases 1, 2, 3 and 4) and was used for standard DNA barcoding analyses with the most comprehensive dataset (we underlined that this dataset contains also sequences derived from morphologically undetermined organisms. *coxI *dataset B includes 168 sequences 630 bp long (gaps are taken into account).

Finally, two datasets identified as as type C (one relative to *coxI *and one relative to 12S rDNA) encompass sequences derived from organisms belonging to cases 1 and 2 and for which both genes were available. These two types of C datasets were used to compare the molecular identification performance of different markers, and of different data handling. The two datasets C include 86 sequences (*coxI *are 627 bp long; 12S rDNA are 643 bp long including gaps) representing 44 morpho-species (with an average of 2.0 specimens per species; standard deviation 1.5; range: 1–6).

### DNA barcoding analyses

In order to evaluate the performance of the DNA barcoding approach performed on filarioid nematodes, the degree of correlation between the species identification based on morphology and on molecular divergences was measured. This test was developed for the mitochondrial gene *coxI *on the widest molecular dataset of filarioid nematodes identified by morphological experts (dataset A).

Intraspecific, interspecific, overall mean K2P distances [28] and relative standard errors were calculated with MEGA 4.1 [29] – options = Kimura 2-parameters, pairwise deletion.

Typical DNA barcoding analyses are based on the comparison between intraspecific and interspecific distribution of nucleotide divergence that allow the inference of a molecular threshold to help taxonomic decision. Based on this approach two kinds of error can occur. Type I errors (false positive) occur when co-specific specimens show a genetic distance greater than threshold value. In contrast, type II errors (false negative) occur when genetic distance minor to the threshold value is found between different species. Cumulative error plots show the error rates generated by both type I and type II errors based on different values of threshold [14]. In this context, the threshold value relative to the minor rate of cumulative error is called optimum threshold (OT). When not a single value, but a range of threshold values is relative to the same minimum cumulative error, the formal OT is calculated as the average value of the range. Differently, a standard threshold (ST) value was calculated as 10 times the mean intraspecific variability according to Hebert *et al*. [11]. Cumulative error rates relative to ST and OT were also compared.

### DNA taxonomy analyses

According to Lefebure *et al*. [8] we will refer to the terms DNA barcoding and DNA taxonomy respectively for: 1) identification of organisms based on DNA sequence variability and assignment to a certain species previously described; 2) prediction and classification of new taxa using DNA.

On the bases of the results obtained with dataset A, the OT generated has been used to perform DNA barcoding and DNA taxonomy approaches on dataset B.

The resulting K2P distance matrix has been used: 1) to infer MOTUs delimited by OT; 2) to analyse the MOTU composition testing the congruence with previously described species (DNA barcoding); 3) to perform prediction and classification of potentially new taxa (DNA taxonomy).

A phenetic tree was also generated for type B dataset of *coxI *marker using MEGA 4.0 [29] – options = tree inference method: neighbor-joining; phylogeny test and options: bootstrap (100 replicates); gaps/missing data: pairwise deletion; codon positions: 1st+2nd+3rd+noncoding; substitution model: K2P; substitutions to include: transitions + transversions; pattern among lineages: same (homogeneous); rates among sites: uniform rates. MOTUs previously identified from K2P distance matrix have been showed on the tree with squared brackets.

### Differential performance of DNA barcoding

Different works show the importance of a proper data management (from the choice of alignment software to the gap treatment) in the context of DNA barcoding analyses (see for instance [30]). The relevance of a DNA barcoding approach based on multiple marker is also underlined by different authors [7,8].

In this work we compared the performance of DNA barcoding performed with different combination of data handling and with different DNA barcodes. The performance comparison was based on the measure of the strength of correlation between morphological and molecular approaches (cumulative error rates were compared).

In this connection homologous DNA sequences of *coxI *and 12S rDNA type C datasets were aligned with two different multiple alignment software: MUSCLE [31] – default options – and ClustalX [32] – default options. Alignments were hand corrected with BioEdit [33] in order to discard the terminal misalignments and were pruned to 627 bp for *coxI *and 643 bp for 12S rDNA (gaps included). K2P distances [28] were calculated with two different applications, and the gaps were treated in two different ways: MEGA [29] – options = Kimura 2-parameters, both pairwise deletion and complete deletion were set in separate runs – and TREECON [34] – options = Kimura 2-parameters, both 'not take into account' and 'take into account' were set in separate runs. K2P distance graph and cumulative error plots were produced for the sixteen combination of software/parameters tested on two type C datasets. A schematic representation of the different approaches used is illustrated in Table 2. Intraspecific, interspecific, overall mean K2P distances [28] and relative standard errors were calculated for *coxI *and 12S rDNA datasets (after alignment with MUSCLE) with MEGA 4.1 [29] – options = Kimura 2-parameters, pairwise deletion.

**Table 2:** Minimum cumulative errors (MCE). Minimum cumulative errors relative to standard threshold (MCE_ST_) and optimum threshold (MCE_OT_) for different markers and different data handling.

**marker**	**alignment**	**distance calculation**	gap treatment*	**ST**	**MCE_ST _(%)**	**OT**	**MCE_OT _(%)**
*coxI*	MUSCLE	TREECON	TA	4.6	0.3	4.5	0.3
*coxI*	MUSCLE	TREECON	NTA	4.6	0.3	4.5	0.3
*coxI*	MUSCLE	MEGA	PD	4.6	0.3	3.9	0.3
*coxI*	MUSCLE	MEGA	CD	4.2	0.3	4.5	0.3
*coxI*	Clustal	TREECON	TA	4.6	0.3	4.5	0.3
*coxI*	Clustal	TREECON	NTA	4.6	0.3	4.5	0.3
*coxI*	Clustal	MEGA	PD	4.6	0.3	3.9	0.3
*coxI*	Clustal	MEGA	CD	4.2	0.3	4.5	0.3
12S rDNA	MUSCLE	TREECON	TA	34.7	99.9	9.0	0.4
12S rDNA	MUSCLE	TREECON	NTA	22.0	88.7	6.4	0.3
12S rDNA	MUSCLE	MEGA	PD	22.0	89.9	6.7	0.3
12S rDNA	MUSCLE	MEGA	CD	12.0	54.7	5.8	1.9
12S rDNA	Clustal	TREECON	TA	22.0	33.5	7.2	0.4
12S rDNA	Clustal	TREECON	NTA	14.3	14.5	5.8	0.4
12S rDNA	Clustal	MEGA	PD	14.3	14.8	5.8	0.4
12S rDNA	Clustal	MEGA	CD	8.1	16.2	4.4	1.1

*NTA is for not taken into account; TA is for taken into account; PD is for pairwise deletion, CD is for complete deletion

## Results

### Morphological identification

A total of 89 specimens collected from 21 localities have been analysed by morphological experts. 76 specimens have been identified as 28 morpho-species, belonging to 12 genera, while 11 specimens (forming 5 distinguishable morphological groups) have not been assigned, at this level of the work, to any described species.

### Analyses on the molecular datasets

The datasets generated in this work comprise a total of 254 gene sequences, 141 of which were produced in this study (for details see additional file 1: 'Investigated specimens'). For a total of 11 morphologically identified species, the DNA gene sequences here reported represent the first entries in GenBank.

### DNA barcoding: coherence between molecular and morphological identifications

The multiple alignment of *coxI *gene sequences forming dataset A presents no insertion/deletion (indels). *coxI *mean nucleotide distance within species is 0.5% (standard error: 0.6%; range: 0 – 2.4%); *coxI *mean nucleotide distance between species is 16.2% (standard error: 3.7%; range: 0 – 27.8%); *coxI *overall mean diversity is 16.0% (standard error: 1.0%).

**Figure 1:**
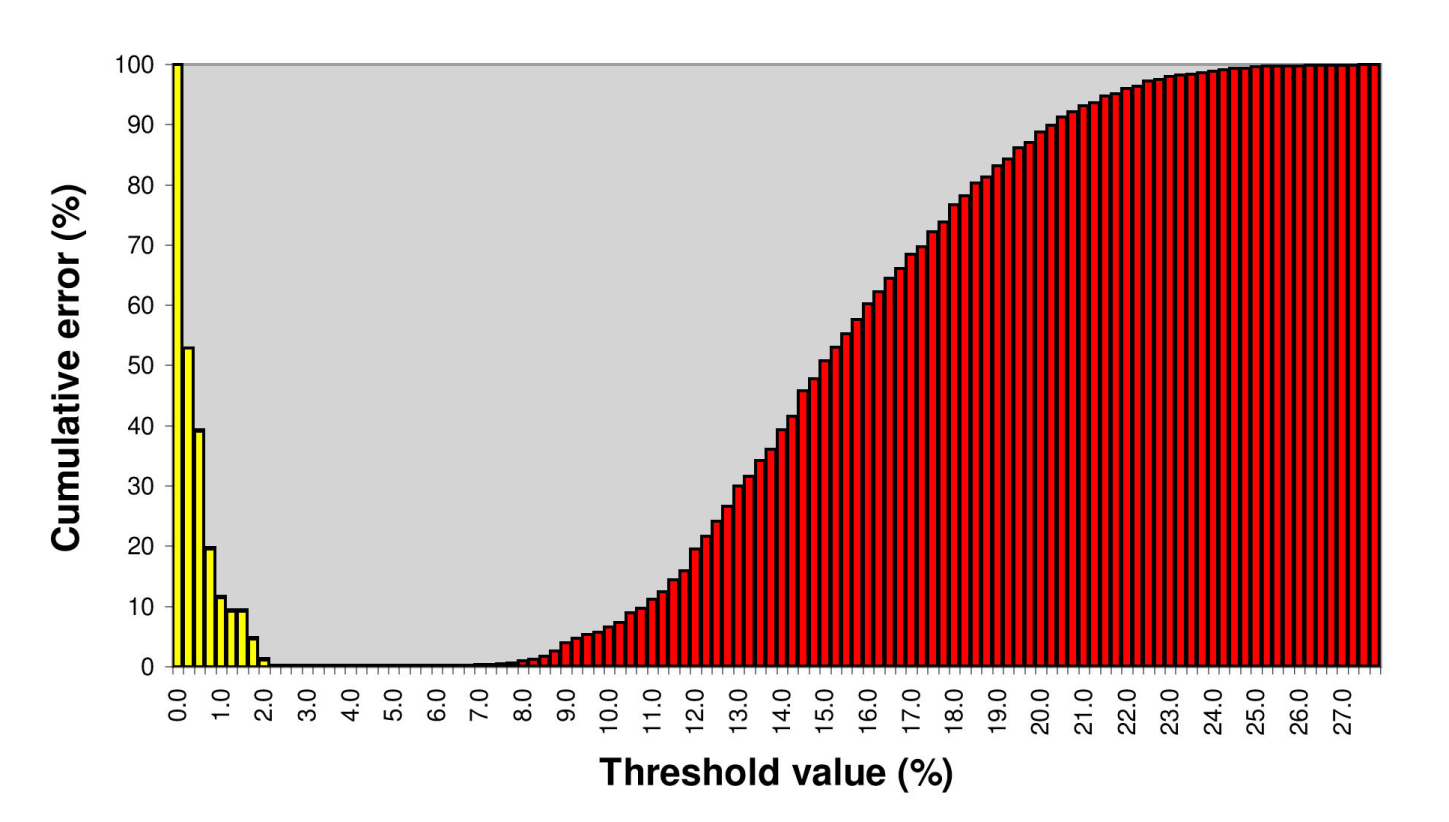
**Cumulative error plot**. Type I (yellow) and type II (red) errors obtained with different thresholds for *coxI *sequences of 46 spirurida species.

Figure 1 shows the frequency distribution of intraspecific and interspecific genetic divergences in *coxI *dataset A. An overlap between the two distributions is observable at values minor to 2%. Since some interspecific divergences are as low as 0% it is not possible to set any threshold value that allow to exclude false negatives (type II errors).

**Figure 2:**
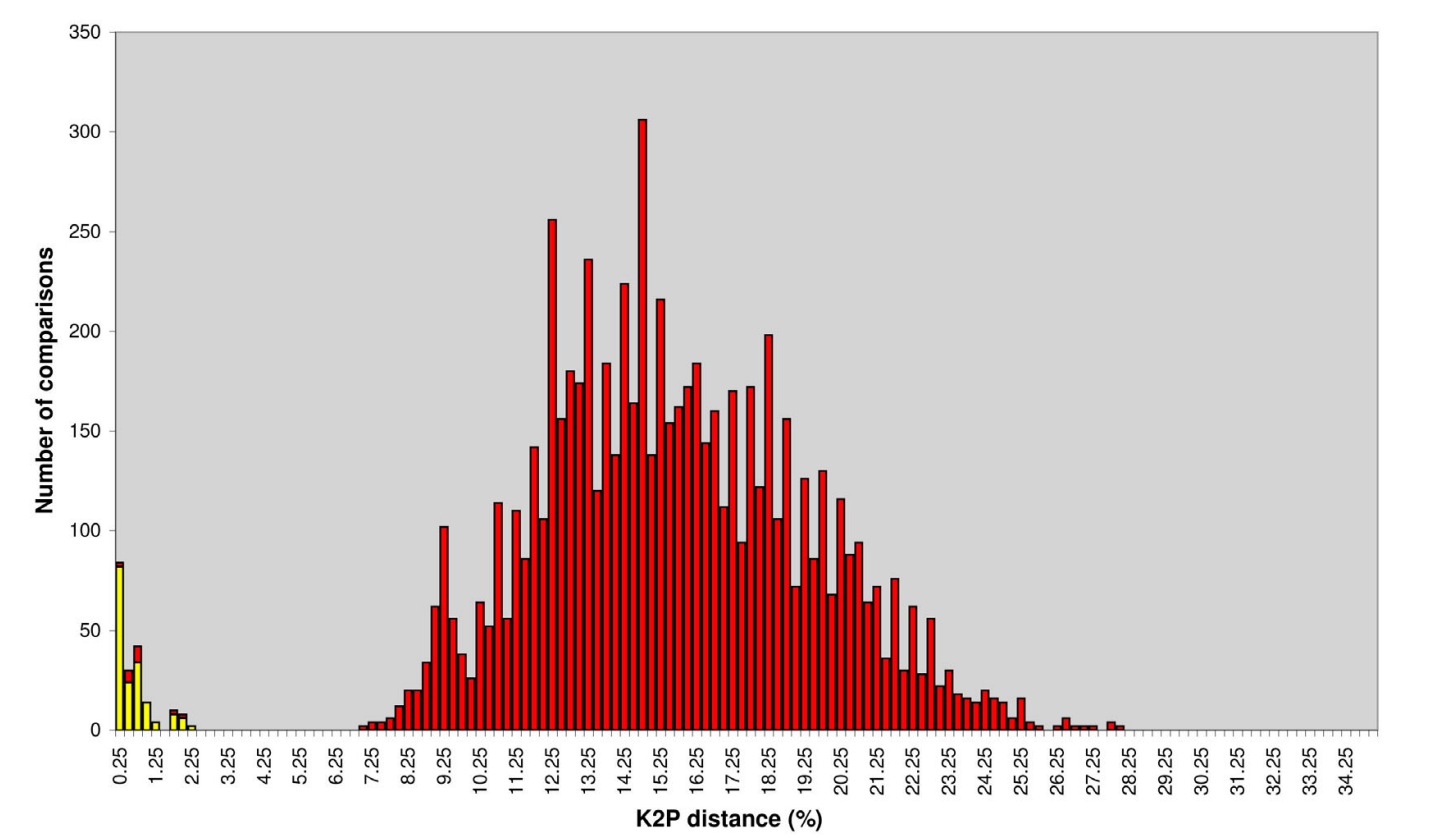
**K2P distance graph of *coxI *filarioid nematodes**. Frequency distribution of intraspecific and interspecific genetic divergences in morphologically identified filarioid nematodes. Graph shows 877 intraspecific and 21775 interspecific comparisons across 46 filarioid species. Distances were generated after alignment with MUSCLE, and calculated with MEGA (pairwise deletion), using Kimura's two parameter substitution model.

The minimum cumulative error is 0.62% (see Figure 1) at a threshold level of 4.8% (OT). ST (10 times intraspecific mean divergence) assumes the value of 5.0% and generates the same cumulative error (0.62%). As shown in K2P distance graph (Figure 2), using OT or ST, no overlap of intraspecific and interspecific nucleotide divergence occurs at distance values greater than threshold values (hence no false positive occur, type I errors). In contrast, as stated before, at distance values lower than OT or ST, a degree of overlap is observable (false negatives, type II errors). This percentage of false negatives (that represents the overall amount of cumulative error) are generated by two couples of congeneric species: 1) *O. volvulus *and *O. ochengi *(mean interspecific distance is 1.9%); 2) *C. bulboidea *and *C. longa *(mean interspecific distance is 0.2%). If *O. volvulus *and *C. bulboidea *are discarded from dataset, no overlap between intraspecific and interspecific distributions are observable, and the OT allows to reach 0% of cumulative error. In summary, identification based on molecular divergence threshold for *coxI *is coherent with morphological approach for 44 species out of 46.

### DNA taxonomy: a direct application of DNA barcoding

Sequences in dataset B (that encompasses all of the *coxI *sequences of filarioid nematodes provided by GenBank) were used to generate a new K2P distance matrix. This has been used to identify the MOTUs whose boundaries are delimited by the OT value (4.8%) calculated at previous point. This approach was used to reach two different purposes: DNA barcoding (MOTUs composition were checked to correspond to previously identified species) and DNA taxonomy (which allow to identify potentially new species). 51 MOTUs were identified: 46 belong to species previously described, and five belong to not morphologically identified organisms (potentially non described species) of filarioid nematodes.

Molecular cryptic species (complexes composed by different morpho-species grouped into single MOTUs) are still present: all of the sequences of *C. bulboidea *and *C. longa *form a single MOTU and all of the sequences of *O. volvulus *and *O. ochengi *form also a single MOTU. This result is concordant with the results obtained from dataset A.

The five unidentified MOTUs encompass specimens collected from avian and mammals hosts. Three out of these contain specimens collected from African mammals (*Oryx gazella*, *Redunca fulvorufula *and *Equus zebra hartmannae*) and the remaining two contain specimens collected from three bird species (*Sitta europea*, *Paradoxornis webbianus *and *Sturnus vulgaris*). Despite a phenetic tree is not the most important output of a DNA barcoding analysis, it can be used to show clearly the pattern of MOTUs composition. For this reason, we provided a NJ tree for dataset B with MOTUs delimited by squared brackets (see additional file 3: 'NJ tree').

### DNA barcoding performance: comparison of different markers and different data handling

Type C datasets were built with sequences deriving from the same specimens in order to allow the comparison of the performances of different markers and different data managements. The performances were evaluated calculating the minimum cumulative error (MCE) rate relative to both optimum threshold and standard threshold values. The lesser the MCE, the better is the performance.

The multiple alignment of *coxI *gene sequences (dataset type C) presents no indels. *coxI *mean nucleotide distance within species is 0.5% (standard error: 0.5%; range: 0 – 2.0%); *coxI *mean nucleotide distance between species is 15.5% (standard error: 3.7%; range: 0.2 – 27.8%); *coxI *overall mean diversity is 15.0% (standard error: 1.0%).

As expected, the multiple alignment of 12S rDNA gene sequences (dataset type C) shows several indels, which were in most cases concentrated in the variable regions. 12S rDNA mean nucleotide distance within species is 2.2% (standard error: 1.7%; range: 0 – 6.0%); 12S rDNA mean nucleotide distance between species is 17.4% (standard error: 4.2%; range: 0.2 – 34.5%); 12S rDNA overall mean diversity is 17.0% (standard error: 1.1%).

Based on MCE (relative to OT) rate comparison, the two DNA barcodes used show different performances (mean MCE for *coxI *is 0.3% and mean MCE for 12S rDNA is 0.7%).

Using the marker *coxI*, the eight different combinations of data handling show the same value of MCE indicating that the performance of DNA barcoding with the marker *coxI *is not susceptible to the tested data handling. OT and ST assume very similar values, and the rates of MCE relative to the different thresholds are the same (see Table 2).

Differently, using 12S rDNA, the eight different combinations of data handling show rates of MCE remarkably different indicating that the performance of DNA barcoding with the marker 12S rDNA is very susceptible to different data handling. With this marker, OT and ST assume very different values, and the performance of DNA barcoding with the two thresholds is extremely dissimilar (mean MCE relative to OT is 0.7%; mean MCE relative to ST is 50.5%).

Interestingly, the two markers show very different manageability: *coxI *has revealed to be less susceptible than 12S rDNA to changes in alignment algorithm, software used for distance estimation, and gap treatment. The lower manageability observed for the marker 12S rDNA is certainly caused by the presence of numerous indels.

Also for datasets C, the errors of DNA barcoding performed with the better data handling are all attributable to false negatives and are relative to the couples of species: *O. volvulus *and *O. ochengi*; *C. bulboidea *and *C. longa*.

## Discussion

On type A dataset a really good discrimination level is achievable, with 44 species out of 46 identifiable via DNA barcoding. For two couples of species, the interspecific divergence is less than the optimal threshold and hence two morpho-species of filarioid nematodes are not resolved by DNA barcoding approach. These two species belong to *Onchocerca *and *Cercopithifilaria *genus. Despite *O. volvulus *and *O. ochengi *are easily identified based on morphology and host specificity, their nucleotide divergence is quite low (mean interspecific divergence 1.9%). If *O. volvulus *infects human patients only in Africa (originally) and South America (following the transatlantic slave trade) and *O. ochengi *infects only cattles, the two species could derive from a recent speciation event [35]. This event could decrease the resolution power of DNA barcoding.

Another putative recent speciation has been proposed for two species of *Cercopithifilaria *genus (*C. longa *and *C. bulboidea*), showing a mean interspecific divergence of 0.2%. These parasites are restricted to two Japanese mammals (*Naemorhaedus crispus *and *Cervus nippon*), and a recent speciation event has also been hypothesized using both molecular and morphological data [27,36]. It should be noted that these evolutionary dynamics are often difficult to identify as reported in [37].

Dataset B (that encompasses all the *coxI *sequences of filarioid nematodes available in GenBank) has been used to perform DNA barcoding and DNA taxonomy with a tree-based method. Coherently with the results obtained with dataset A, this phenetic approach shows a clear separation of MOTUs representing separated groups of morpho-species with the exception of *O. volvulus*-*O. ochengi *and *C. bulboidea*-*C. longa*. Anyway, closely related species could be characterized by a certain level of interspecific hybridization, because the reproductive isolation could not be total since the very beginning of the natural history of a species. These effects are particularly evident in mitochondrial gene trees, and represent a serious problem for DNA barcoding (at least in most metazoans, for which mitochondrial markers are widely used). Problems of this nature are likely to have occurred in the *O. volvulus*-*O. ochengi *and *C. bulboidea*-*C. longa *cases where traditional taxonomy identified good species [27,38]. As a consequence, the usage of a tree-based method alone for species identification could be dangerous and deceptive. Moreover, in a gene tree, a 'true' species may be wrongly represented by a paraphyletic group of alleles/haplotypes, due to introgression or incomplete lineage sorting (see [15]). In such cases, the gene tree could appear misleading or uninformative about the species identification because of retention, and consequent random sorting, of ancestral polymorphisms.

It is important to underline that GenBank entries are not absolutely free from identification errors. The results of DNA barcoding analyses performed on *coxI *sequences obtained from GenBank (dataset B) do not show such type of problem. However, an example of error is represented by the entry [GenBank:AY462911] identified as *Litomosoides carinii*. This species parasites sciurids in Brazil [39] and was described by Travassos in 1916. The congeneric species *Litomosoides sigmodontis *was described by Chandler in 1931, parasites the murid *Sigmodon hispidus*, and is spread worldwide in the laboratories as model species for the studies on filarioses. For some reasons there is the tendency to confound these two clearly distinct species, and it is relatively common to observe the erroneous name *L. carinii *used instead of *L. sigmodontis *for laboratory strains of these filariods. In this context, it should be noted that basically all the results on *L. sigmodontis *published till now are relative to these laboratory strains established since 1970s. Here we present a molecular identification of *L. sigmodontis *directly collected from wild hosts. Laboratory strains and wild specimens show no molecular differences.

The five unidentified MOTUs present in dataset B encompass parasites of three avian hosts, a taxonomic group where biodiversity and distribution of filarial nematodes are underestimated. As described above, these are cases where molecular analysis can help to discover new species (DNA taxonomy).

It must be underlined that DNA taxonomy performed with simple molecular data can only suggest the presence of potential new species, whose real existence must be corroborated by integrated approaches [40].

Type C datasets reveal that two different markers have similar discrimination power, but if *coxI *shows high manageability in data handling, the marker 12S rDNA is more susceptible to the data handling (especially in gap treatment). Processing 12S rDNA type C dataset with MUSCLE and MEGA (pairwise deletion), DNA barcoding performs 6.3 time better than using MUSCLE and MEGA (complete deletion). In addition, processing 12S rDNA type C dataset with MUSCLE it is possible to obtain 0.3% of MCE (see Table 2), whilst using ClustalX, it is possible to obtain 0.4% of MCE (see Table 2). This is a quite relevant observation: the generation of a reliable alignment is a major impediment limiting the use of 12S rDNA gene sequences for barcoding purposes. For this reason, Chu et al. [41] have proposed to use ribosomal DNA sequences for DNA barcoding without performing an alignment, showing congruence between their approach and a tree reconstruction (based on neighbour-joining algorithm). Anyway, 12S rDNA offers practical benefits: it is much shorter compared with *coxI*, and therefore more likely to be readily amplified from chemically damaged (i.e. formalin fixed) or badly conserved specimens [9].

It is important to underline that the presence of nuclear mitochondrial pseudogenes (numts [42]) could introduce serious ambiguity into DNA barcoding and their presence cannot be known *a priori *[43]. In nematodes, numts seems to be rare [42], despite their presence has been reported (see for example [44] were a short fragment of the mitochondrial 16S rDNA of *W. bancrofti *included into the nuclear LDR region is used for the screening of this parasite). In our study, the results of BLAST search, multiple alignment analyses and the quality of trace files for bidirectional processing of our sequences seems to exclude any interference caused by numts.

Our results indicate that the proposal to use the ST (10 times intraspecific variability) as described in [11] must be evaluated case by case. Indeed, in the case of *coxI*, the OT is equivalent to ST (both the thresholds generate the same value of MCE), but for 12S rDNA OT performs extremely better than ST (mean MCE relative to OT is 0.7%, mean MCE relative to ST is 50.5%). The extremely high values of MCE relative to ST are caused by the moderately high intraspecific K2P distances of the marker 12S rDNA that are enhanced of a 10 times magnitude. The data handling has also a relevant effect on the mean intraspecific divergence: MUSCLE, TREECON and considering gaps are all alternatives that enhance K2P distances.

The sampling of filarioid nematodes is clearly not exhaustive and particularly difficult, due to complications associated with their collection (i.e. recovery at necropsy in most of the cases), that requires highly skilled personnel and enduring logistic efforts all over the world. The datasets presented encompass also species for which only one sequence is available. This is a circumstance that avoid to evaluate the intraspecific variability of the marker, and consequently the discrimination power of the method decreases. However, we want to remark the importance of the datasets here reported: filarioid nematodes represent a relevant neglected, vector-borne, tropical diseases.

## Conclusion

DNA barcoding represents a powerful tool for taxonomy, but without the integration of traditional approaches could become a simple collection of MOTUs. Recent studies showed that different approaches to species recognition can generate similar results, encountering the favour of scientific community [45] suggesting that an integrated approach to species recognition is a possibility [46]. In our opinion, the establishment, improvement and maintenance of DNA barcoding as a taxonomic tool will require a long-lasting interaction between traditional taxonomy and DNA-based approaches. In this work traditional and molecular approaches have been considered as an integrated method for achieving the goal of species identification.

DNA barcoding is a good method for taxonomical identification of filarioid nematodes, and it has shown a high coherence with classical taxonomy. The results of the integrated approach to species identification clearly show where DNA-based and morphological identifications are consistent, and where they are not.

This study suggests that both *coxI *and 12S rDNA appear to be appropriate molecular markers for identification of filarioid nematodes at species level via DNA barcoding. More in detail, the results of DNA barcoding has been shown to be more consistent under different data handling when performed with *coxI *than 12S rDNA. On the opposite, 12S rDNA is less manageable, but it is easier to amplify than *coxI*.

The threshold value proposed by Hebert (10 times mean intraspecific divergence) [1] has revealed to be applicable for *coxI*, and not for 12S rDNA. In the case of *coxI*, the threshold value 4.8% can also be used to separate potentially new filarioid species. We conclude that nucleotide sequences of *coxI *from filarioids are of high interest for species identification throughout DNA barcoding. Despite the databases here reported encompass only few specimens of medical interests, they represent a useful starting point for rapid identification of these parasites and for applications such as epidemiological surveys and populational dynamics.

Using *coxI *with a threshold that minimise the error rate, all of the filarial nematodes involved in medical or veterinarian context (with the exception of *O. volvulus *and *O. ochengi*) can be coherently identified as morphological (species) and molecular entities (MOTUs). Finally, it should be noted that the two approaches for species identification (i.e. morphological and molecular) are not consistent at 100%. This is not unexpected, both methods are susceptible to different bias: sibling species, morphological polymorphisms, introgression and coalescence can, for instance, confound identifications. One way to seriously cope with these difficulties is to follow the cross control given by different approaches (for works about integrated taxonomy see [8,40]). The possibility to identify how and where the different approaches are not coherent can be the first step for developing of a true integrated approach to taxonomy.

## Competing interests

The authors declare that they have no competing interests.

## Authors' contributions

EF is a post-doctoral researcher interested in bioinformatics and developed the analyses. MB and AG performed PCRs and sequencing. OB, SU, RG and HF collected and identified most of the samples used in the analyses using traditional taxonomy. CM and CB highly contributed to the discussion of the results. CB was also essential in the initial contact between the researchers that originated this international research network. MC played the role of inspirer and coordinator of the research.

## Supplementary Material

Additional File 1 **Investigated specimens**. **List of species including biological data, accession numbers and datasets (for data analysis) of the nematodes included in this study.** Where available date, place of collection and hosts are indicated (n.d.: no data available). * The host species indicated as *Naemorhedus crispus *is synonymous to *Capricornis crispus*. ** Laboratory strain in European laboratories since 1970s. *** Collected from a patient travelling from Camerun. **** Collected from a patient travelling from India. Click here for file

Additional File 2 **Experimental conditions**. **Detailed conditions of DNA extraction, primers, PCRs and DNA sequencing.** Click here for file

Additional File 3 **NJ Tree. **Neighbour joining tree based on *coxI *sequences generated using MEGA 4.0 (Tamura *et al*, 2007) – gaps treated as 'complete deletion'. Click here for file
