# Supplementary material for: Integrated taxonomy: traditional approach and DNA barcoding for the identification of filarioid worms and related parasites (Nematoda)
Source: Front Zool. 2009 Jan 7;6:1. doi: 10.1186/1742-9994-6-1 (PMC2657783; doi:10.1186/1742-9994-6-1)
Supplement: Additional File 1 — Investigated specimens. List of species including biological data, accession numbers and datasets (for data analysis) of the nematodes included in this study. Where available date, place of collection and hosts are indicated (n.d.: no data available). * The host species indicated as Naemorhedus crispus is synonymous to Capricornis crispus. ** Laboratory strain in European laboratories since 1970s. *** Collected from a patient travelling from Camerun. **** Collected from a patient travelling from India. [file 1742-9994-6-1-S1.pdf]

Species/MOTU	ID Number	Voucher	Accession number coxI	Accession number 12s rDNA	Dataset	Host	Data	Collection place	Produced in this study
Acanthocheilonema reconditum (Grassi, 1890)	-	-	AJ544876	AJ544853	A,B,C	*Canis lupus familiaris*	-	-	-
Acanthocheilonema viteae (Krepkogorskaya, 1933)	-	-	AJ272117	AJ544852	A,B,C	*Meriones libycus*	-	-	-
Brugia malayi (Brug, 1927)	-	-	AJ271610	AJ544843	A,B,C	*Homo sapiens*	-	-	-
Brugia malayi (Brug, 1927)	-	-	AF538716	AF538716	A,B,C	n.d.	-	-	-
Brugia pahangi (Buckley & Edeson, 1956)	-	-	EF406112	-	A,B	*Homo sapiens*	-	-	-
Brugia pahangi (Buckley & Edeson, 1956)	-	-	DQ977746	-	B	*Meriones unguiculatus*	-	-	-
Brugia pahangi (Buckley & Edeson, 1956)	-	-	AJ271611	AJ544842	A,B,C	*Felis catus*	-	-	-
Cercopithifilaria bulboidea Uni & Bain, 2001	C1-3	MIB:Zpl:00940	AM749247	AM779779	A,B,C	*Naemorhedus crispus* *	25/12/2004	Japan, Gifu	YES
Cercopithifilaria bulboidea Uni & Bain, 2001	C1-4A	MIB:Zpl:00942	AM749248	AM779780	A,B,C	*Naemorhedus crispus* *	25/12/2004	Japan, Gifu	YES
Cercopithifilaria bulboidea Uni & Bain, 2001	Isolate Gifu-111	-	AB178834	-	A,B	*Capricornis crispus*	-	-	-
Cercopithifilaria bulboidea Uni & Bain, 2001	Isolate Gifu-133	-	AB178835	-	A,B	*Capricornis crispus*	-	-	-
Cercopithifilaria bulboidea Uni & Bain, 2001	Isolate SW3-FL7	-	AB178836	-	A,B	*Capricornis crispus*	-	-	-
Cercopithifilaria bulboidea Uni & Bain, 2001	Isolate SW3-FL8	-	AB178837	-	A,B	*Capricornis crispus*	-	-	-
Cercopithifilaria bulboidea Uni & Bain, 2001	Isolate SW3-MB1	-	AB178838	-	A,B	*Capricornis crispus*	-	-	-
Cercopithifilaria bulboidea Uni & Bain, 2001	Isolate SW3-UA1	-	AB178839	-	A,B	*Capricornis crispus*	-	-	-
Cercopithifilaria crassa Uni, Bain & Takaoka, 2002	Isolate S15-097	-	AB178840	-	A,B	*Cervus nippon*	-	-	-
Cercopithifilaria crassa Uni, Bain & Takaoka, 2002	Isolate S15-101	-	AB178841	-	A,B	*Cervus nippon*	-	-	-
Cercopithifilaria crassa Uni, Bain & Takaoka, 2002	S51-PB6	MIB:Zpl:00925	AM749260	AM779791	A,B,C	*Cervus nippon*	18/02/2003	Japan, Oita	YES
Cercopithifilaria japonica (Uni, 1983)	BS6-2	MIB:Zpl:01156	AM749261	AM779794	A,B,C	*Ursus thibetanus*	06/12/2004	Japan, Gifu	YES
Cercopithifilaria japonica (Uni, 1983)	BS9-1	MIB:Zpl:00941	AM749262	AM779793	A,B,C	*Ursus thibetanus*	01/12/2004	Japan, Gifu	YES
Cercopithifilaria japonica (Uni, 1983)	BP5-1	MIB:Zpl:00939	AM749263	AM779792	A,B,C	*Ursus thibetanus*	01/12/2004	Japan, Gifu	YES
Cercopithifilaria longa Uni, Bain & Takaoka, 2002	S51-PB1	MIB:Zpl:00926	AM749243	AM779783	A,B,C	*Cervus nippon*	10/02/2003	Japan, Oita	YES
Cercopithifilaria longa Uni, Bain & Takaoka, 2002	AG1-10	MIB:Zpl:00931	AM749244	AM779782	A,B,C	*Cervus nippon*	03/07/2003	Japan, Hyogo	YES
Cercopithifilaria longa Uni, Bain & Takaoka, 2002	AG1-5	MIB:Zpl:00930	AM749245	AM779781	A,B,C	*Cervus nippon*	03/07/2003	Japan, Hyogo	YES
Cercopithifilaria longa Uni, Bain & Takaoka, 2002	Isolate S32-2	-	AB178842	-	A,B	*Cervus nippon*	-	-	-
Cercopithifilaria longa Uni, Bain & Takaoka, 2002	Isolate S32-4	-	AB178843	-	A,B	*Cervus nippon*	-	-	-
Cercopithifilaria longa Uni, Bain & Takaoka, 2002	Isolate S33-4	-	AB178844	-	A,B	*Cervus nippon*	-	-	-
Cercopithifilaria longa Uni, Bain & Takaoka, 2002	Isolate S33-6	-	AB178845	-	A,B	*Cervus nippon*	-	-	-
Cercopithifilaria longa Uni, Bain & Takaoka, 2002	S51-PB2	MIB:Zpl:00912	AM749246	AM779784	A,B,C	*Cervus nippon*	11/02/2003	Japan, Oita	YES
Cercopithifilaria minuta Uni & Bain 2001	C1-A4	MIB:Zpl:00905	AM749252	AM779785	A,B,C	*Naemorhedus crispus* *	05/05/2003	Japan, Yamagata	YES
Cercopithifilaria minuta Uni & Bain 2001	SW1-23	MIB:Zpl:00915	AM749253	AM779786	A,B,C	*Naemorhedus crispus* *	07/08/2002	Japan, Yamagata	YES
Cercopithifilaria minuta Uni & Bain 2001	Isolate SW3-FL3	-	AB178846	-	A,B	*Capricornis crispus*	-	-	-
Cercopithifilaria minuta Uni & Bain 2001	Isolate SW3-FL12	-	AB178847	-	A,B	*Capricornis crispus*	-	-	-
Cercopithifilaria multicauda Uni & Bain, 2001	G119	MIB:Zpl:00921	AM749255	AM779799	A,B,C	*Naemorhedus crispus* *	26/08/1999	Japan, Gifu	YES
Cercopithifilaria multicauda Uni & Bain, 2001	SW3-FL9	MIB:Zpl:00922	AM749254	AM779800	A,B,C	*Naemorhedus crispus* *	10/05/2001	Japan, Gifu	YES
Cercopithifilaria multicauda Uni & Bain, 2001	Isolate Gifu-39T	-	AB178848	-	A,B	*Capricornis crispus*	-	-	-
Cercopithifilaria multicauda Uni & Bain, 2001	Isolate Gifu-49C	-	AB178849	-	A,B	*Capricornis crispus*	-	-	-
Cercopithifilaria roussilhoni Bain, Petit & Chabaud, 1986	143 SE	MIB:Zpl:00959	AM749264	AM779798	A,B,C	*Atherurus africanus*	1995	Gabon, Makokou Station	YES
Cercopithifilaria shohoi Uni, Suzuki & Katsumi, 1998	C1-LB4	MIB:Zpl:00906	AM749249	AM779795	A,B,C	*Naemorhedus crispus* *	06/05/2003	Japan,Yamagata	YES
Cercopithifilaria shohoi Uni, Suzuki & Katsumi, 1998	SW1-32	MIB:Zpl:00919	AM749250	AM779796	A,B,C	*Naemorhedus crispus* *	08/08/2002	Japan,Yamagata	YES
Cercopithifilaria shohoi Uni, Suzuki & Katsumi, 1998	SW21-170	MIB:Zpl:00923	AM749251	AM779797	A,B,C	*Naemorhedus crispus* *	21/05/1999	Japan,Yamagata	YES
Cercopithifilaria shohoi Uni, Suzuki & Katsumi, 1998	Isolate Gifu-07	-	AB178850	-	A,B	*Capricornis crispus*	-	-	-
Cercopithifilaria shohoi Uni, Suzuki & Katsumi, 1998	Isolate Gifu-14	-	AB178851	-	A,B	*Capricornis crispus*	-	-	-
Cercopithifilaria tumidicervicata Uni & Bain, 2001	C1-LB8	MIB:Zpl:00904	AM749256	AM779788	A,B,C	*Naemorhedus crispus* *	06/05/2003	Japan,Yamagata	YES
Cercopithifilaria tumidicervicata Uni & Bain, 2001	SW5-119	MIB:Zpl:00914	AM749257	AM779790	A,B,C	*Naemorhedus crispus* *	26/04/1999	Japan,Yamagata	YES
Cercopithifilaria tumidicervicata Uni & Bain, 2001	C1-LBB1	MIB:Zpl:00901	AM749258	AM779787	A,B,C	*Naemorhedus crispus* *	07/05/2003	Japan,Yamagata	YES
Cercopithifilaria tumidicervicata Uni & Bain, 2001	SW1-9	MIB:Zpl:00918	AM749259	AM779789	A,B,C	*Naemorhedus crispus* *	06/08/2002	Japan,Yamagata	YES
Cercopithifilaria tumidicervicata Uni & Bain, 2001	Isolate Gifu-91	-	AB178852	-	A,B	*Capricornis crispus*	-	-	-
Cercopithifilaria tumidicervicata Uni & Bain, 2001	Isolate Gifu-132	-	AB178853	-	A,B	*Capricornis crispus*	-	-	-
Dipetalonema gracile (Rudolphi, 1809)	-	-	AJ544877	AJ544854	A,B,C	*Cebus olivaceus*	-	-	-
Dipetalonema gracile (Rudolphi, 1809)	15YU	MIB:Zpl:01175	AM749279	AM779824	A,B,C	*Cebus olivaceus*	2004	Venezuela, Yutaje	YES
Dirofilaria (Dirofilaria) immitis (Leidy, 1856)	-	-	DQ358815	-	B	*Canis lupus*	-	-	-
Dirofilaria (Dirofilaria) immitis (Leidy, 1856)	-	-	AJ271613	AJ544831	A,B,C	*Canis lupus familiaris*	-	-	-
Dirofilaria (Dirofilaria) immitis (Leidy, 1856)	-	-	AJ537512	AJ537512	A,B,C	*Canis lupus familiaris*	-	-	-
Dirofilaria (Dirofilaria) immitis (Leidy, 1856)	CATR	MIB:Zpl:01165	AM749226	AM779769	A,B,C	*Felis catus*	2003	Italy, Milan	YES
Dirofilaria (Dirofilaria) immitis (Leidy, 1856)	GEN2	MIB:Zpl:01170	AM749227	AM779771	A,B,C	*Felis catus*	2003	Italy, Milan	YES
Dirofilaria (Dirofilaria) immitis (Leidy, 1856)	GEN3	MIB:Zpl:01167	AM749228	AM779770	A,B,C	*Canis lupus familiaris*	2002	Italy, Milan	YES
Dirofilaria (Dirofilaria) immitis (Leidy, 1856)	M1	MIB:Zpl:01157	AM749229	-	A,B	*Canis lupus familiaris*	2002	Italy, Milan	YES
Dirofilaria (Dirofilaria) immitis (Leidy, 1856)	-	-	EU159111	-	A,B	*Canis lupus familiaris*	-	-	-
Dirofilaria (Nochtiella) repens Railliet & Henry, 1911	-	-	DQ358814	-	B	*Canis lupus*	-	-	-
Dirofilaria (Nochtiella) repens Railliet & Henry, 1911	-	-	AJ271614	AJ544832	A,B,C	*Canis lupus familiaris*	-	-	-
Dirofilaria (Nochtiella) repens Railliet & Henry, 1911	CATM	MIB:Zpl:01166	AM749232	AM779777	A,B,C	*Felis catus*	2001	Italy, Milan	YES
Dirofilaria (Nochtiella) repens Railliet & Henry, 1911	PAV	MIB:Zpl:01148	AM749233	AM779774	A,B,C	*Homo sapiens*	2006	Italy, Pavia	YES
Dirofilaria (Nochtiella) repens Railliet & Henry, 1911	POZ	MIB:Zpl:01163	AM749234	AM779778	A,B,C	*Homo sapiens*	2001	Italy, Rome	YES
Dirofilaria (Nochtiella) repens Railliet & Henry, 1911	CATG	MIB:Zpl:01168	AM749231	AM779773	A,B,C	*Felis catus*	2003	Italy, Milan	YES
Dirofilaria (Nochtiella) repens Railliet & Henry, 1911	GEN1	MIB:Zpl:01171	AM749230	AM779775	A,B,C	*Canis lupus familiaris*	2002	Italy, Milan	YES
Filaria martis Gmelin, 1790	-	-	AJ544880	AJ544855	A,B,C	*Martes foina*	-	-	-
Foleyella furcata (Linstow, 1899)	-	-	AJ544879	AJ544841	A,B,C	*Chameleon (not determined)*	-	-	-
Litomosa westi (Gardner & Smith, 1986)	-	-	AJ544871	AJ544851	A,B,C	*Geomys bursarius*	-	-	-
Litomosoides brasiliensis Lins de Almeida, 1936	-	-	AJ544867	AJ544850	A,B,C	*Carollia perspicillata*	-	-	-
Litomosoides galizai Bain, Petit, Diagne, 1989	-	-	AJ544870	AJ544849	A,B,C	*Oecomys tr. tapajinus*	-	-	-

Litomosoides hamletti Sandground, 1934	-	-	AJ544868	AJ544847	A,B,C	*Glossophaga soricina*	-	-	-
Litomosoides scotti Forrester & Kinsella, 1973	-	-	EF661995	-	B	*n.d.*	-	-	-
Litomosoides sigmodontis Chandler, 1931	-	-	AJ271615	AJ544848	A,B,C	*Sigmodon hispidus*	-	France, Paris **	-
Litomosoides sigmodontis Chandler, 1931	1L	MIB:Zpl:01164	AM749286	AM779834	A,B,C	*Sigmodon hispidus*	2005	Venezuela, Yutaje	YES
Litomosoides yutajensis Guerrero, Martin & Bain, 2003	39 YU	MIB:Zpl:01155	AM749280	AM779825	A,B,C	*Pteronotus parnellii*	2004	Venezuela, Yutaje	YES
Litomosoides yutajensis Guerrero, Martin & Bain, 2003	-	-	AJ544869	AJ544846	A,B,C	*Pteronotus parnelli*	-	-	-
Loa loa (Cobbold, 1864)	-	-	AJ544875	AJ544845	A,B,C	*Homo sapiens*	-	-	-
Loxodontofilaria caprini Uni & Bain, 2006	YG2-25	MIB:Zpl:00928	AM749237	AM779817	A,B,C	*Naemorhedus crispus* *	24/06/2003	Japan,Yamagata	YES
Loxodontofilaria caprini Uni & Bain, 2006	YG3-1	MIB:Zpl:00946	AM749238	AM779818	A,B,C	*Naemorhedus crispus* *	23/07/2003	Japan,Yamagata	YES
Loxodontofilaria caprini Uni & Bain, 2006	C1-1A	MIB:Zpl:00903	AM749239	AM779820	A,B,C	*Naemorhedus crispus* *	03/05/2003	Japan,Yamagata	YES
Loxodontofilaria caprini Uni & Bain, 2006	C1-SB10	MIB:Zpl:01151	AM749240	AM779821	A,B,C	*Naemorhedus crispus* *	12/05/2003	Japan,Yamagata	YES
Loxodontofilaria caprini Uni & Bain, 2006	YG3-12	MIB:Zpl:01149	AM749241	AM779819	A,B,C	*Naemorhedus crispus* *	01/08/2003	Japan,Yamagata	YES
Loxodontofilaria caprini Uni & Bain, 2006	C1-FFL1	MIB:Zpl:00902	AM749242	AM779822	A,B,C	*Naemorhedus crispus* *	08/05/2003	Japan,Yamagata	YES
Mansonella (Cutifilaria) perforata Uni, Bain & Takaoka, 2004	S51-PB9	MIB:Zpl:00911	AM749265	AM779803	A,B,C	*Cervus nippon*	18/02/2003	Japan, Oita	YES
Mansonella (Tetrapetalonema) atelensis amazonae n. subsp. Bain & Guerrero, 2008	15 YU	MIB:Zpl:00958	AM749278	AM779823	A,B,C	*Cebus olivaceus*	2004	Venezuela, Yutaje	YES
Spirurida sp.MOTU1	CAM7	MIB:Zpl:01160	AM749287	-	B	*Redunca fulvorufula*	03/03/2004	North Cameroon, Daoud Safari	YES
Spirurida sp.MOTU1	CAM8	MIB:Zpl:01158	AM749288	-	B	*Redunca fulvorufula*	03/03/2004	North Cameroon, Daoud Safari	YES
Spirurida sp.MOTU2	NAM5	MIB:Zpl:01162	AM749289	-	B	*Equus zebra hartmannae*	11/05/2003	Namibia, Ohorongo Safari Outjo	YES
Spirurida sp.MOTU2	NAM6	MIB:Zpl:01150	AM749290	-	B	*Equus zebra hartmannae*	11/05/2003	Namibia, Ohorongo Safari Outjo	YES
Spirurida sp.MOTU3	SIT1	MIB:Zpl:00887	AM749291	-	B	*Sitta europea*	09/03/2006	France, Pas-de-Calais, Dpt 62	YES
Spirurida sp.MOTU3	D06	MIB:Zpl:01153	AM749292	-	B	*Paradoxornis webbianus*	10/09/2005	Itay, Palude Brabbia	YES
Spirurida sp.MOTU3	ID07	MIB:Zpl:01152	AM749293	-	B	*Paradoxornis webbianus*	12/09/2005	Italy, Palude Brabbia	YES
Spirurida sp.MOTU4	DIP1	MIB:Zpl:00886	AM749294	-	B	*Sturnus vulgaris*	25/02/2006	France, Roanne, Dpt 42	YES
Spirurida sp.MOTU4	DIP2	MIB:Zpl:00885	AM749295	-	B	*Sturnus vulgaris*	25/02/2006	France,Firminy, Dpt 42	YES
Spirurida sp.MOTU4	DIP3	MIB:Zpl:01154	AM749296	-	B	*Sturnus vulgaris*	25/02/2006	France, St Etienne, Dpt 42	YES
Spirurida sp.MOTU5	NAM7	MIB:Zpl:01161	AM749297	-	B	*Oryx gazella*	14/05/2003	Namibia, Ohorongo Safari Outjo	YES
Ochoterenella sp. *sensu* Casiraghi et al., 2004	-	-	AJ544878	-	B	*Bufo marinus*	-	-	-
Onchocerca dewittei japonica Uni, Bain & Takaoka, 2001	B61-7	MIB:Zpl:00913	AM749266	AM779816	A,B,C	*Sus scrofa leucomystax*	03/07/2003	Japan, Oita	YES
Onchocerca dewittei japonica Uni, Bain & Takaoka, 2001	B61-4	MIB:Zpl:00917	AM749267	AM779815	A,B,C	*Sus scrofa leucomystax*	03/07/2003	Japan, Oita	YES
Onchocerca eberhardi Uni & Bain, 2007	S51-9	MIB:Zpl:00956	AM749268	AM779810	A,B,C	*Cervus nippon*	10/02/2003	Japan, Oita	YES
Onchocerca gibsoni (Cleland & Johnston, 1910)	-	-	AJ271616	AJ544837	A,B,C	*Bos taurus*	-	-	-
Onchocerca lupi Rodonaja, 1967	-	-	AJ415417	-	A,B	*Canis lupus familiaris*	-	-	-
Onchocerca lupi Rodonaja, 1967	-	-	EF521408	-	A,B	*Canis lupus familiaris*	-	-	-
Onchocerca lupi Rodonaja, 1967	-	-	EF521409	-	A,B	*Canis lupus familiaris*	-	-	-
Onchocerca lupi Rodonaja, 1967	-	-	EF521410	-	A,B	*Canis lupus familiaris*	-	-	-
Onchocerca ochengi Bwangamoi, 1969	-	-	AJ271618	AJ544839	A,B,C	*Bos taurus*	-	-	-
Onchocerca skrjabini Ruklyadev, 1964	S51-2	MIB:Zpl:00924	AM749269	AM779804	A,B,C	*Cervus nippon*	09/02/2003	Japan, Oita	YES
Onchocerca skrjabini Ruklyadev, 1964	S51-7	MIB:Zpl:00910	AM749270	AM779806	A,B,C	*Cervus nippon*	10/02/2003	Japan, Oita	YES
Onchocerca skrjabini Ruklyadev, 1964	S51-4	MIB:Zpl:00908	AM749271	AM779805	A,B,C	*Cervus nippon*	09/02/2003	Japan, Oita	YES
Onchocerca skrjabini Ruklyadev, 1964	G30	MIB:Zpl:00916	AM749272	AM779808	A,B,C	*Naemorhedus crispus* *	19/07/1999	Japan, Gifu	YES
Onchocerca skrjabini Ruklyadev, 1964	C1-FL5	MIB:Zpl:00909	AM749273	AM779807	A,B,C	*Naemorhedus crispus* *	08/05/2003	Japan, Yamagata	YES
Onchocerca skrjabini Ruklyadev, 1964	SW30-26	MIB:Zpl:00920	AM749274	AM779809	A,B,C	*Naemorhedus crispus* *	08/02/2001	Japan, Yamagata	YES
Onchocerca suzukii Yagi, Bain & Shoho, 1994	YG2-35	MIB:Zpl:00932	AM749275	AM779811	A,B,C	*Naemorhedus crispus* *	10/06/2003	Japan, Yamagata	YES
Onchocerca suzukii Yagi, Bain & Shoho, 1994	YG2-53	MIB:Zpl:00937	AM749276	AM779813	A,B,C	*Naemorhedus crispus* *	25/06/2003	Japan, Yamagata	YES
Onchocerca suzukii Yagi, Bain & Shoho, 1994	YG2-37	MIB:Zpl:00935	AM749277	AM779812	A,B,C	*Naemorhedus crispus* *	25/06/2003	Japan, Yamagata	YES
Onchocerca volvulus (Leuckart, 1893)	-	-	NC_001861	-	A,B	*Homo sapiens*	-	-	-
Onchocerca volvulus (Leuckart, 1893)	M4	MIB:Zpl:01172	AM749285	AM779855	A,B,C	*Homo sapiens*	2001	Italy, Rome ***	YES
Onchocerca volvulus (Leuckart, 1893)	M3	MIB:Zpl:01173	AM749284	AM779854	A,B,C	*Homo sapiens*	2001	Italy, Rome ***	YES
Piratuba scaffi Bain, 1974	34 YU_1	MIB:Zpl:00955	AM749281	AM779831	A,B,C	*Ameiva ameiva*	2003	Venezuela, Yutaje	YES
Piratuba scaffi Bain, 1974	34 YU_2	MIB:Zpl:00956	AM749282	AM779832	A,B,C	*Ameiva ameiva*	2004	Venezuela, Yutaje	YES
Piratuba scaffi Bain, 1974	34 YU_3	MIB:Zpl:00957	AM749283	-	B	*Ameiva ameiva*	2004	Venezuela, Yutaje	YES
Setaria digitata (Linstow, 1906)	ST1	MIB:Zpl:00936	AM886173	AM779801	A,B,C	*Bos taurus*	08/09/2003	Japan, Yamagata	YES
Setaria digitata (Linstow, 1906)	Isolate SL/2005/K/Sd1	-	EF174428	-	A,B	*Bos taurus*	-	-	-
Setaria digitata (Linstow, 1906)	Isolate SL/2005/K/Sd9	-	EF174427	-	A,B	*Bos taurus*	-	-	-
Setaria digitata (Linstow, 1906)	Isolate SL/2005/K/Sd8	-	EF174426	-	A,B	*Bos taurus*	-	-	-
Setaria digitata (Linstow, 1906)	Isolate SL/2005/K/Sd7	-	EF174425	-	A,B	*Bos taurus*	-	-	-
Setaria digitata (Linstow, 1906)	Isolate SL/2005/K/Sd6	-	EF174424	-	A,B	*Bos taurus*	-	-	-
Setaria digitata (Linstow, 1906)	Isolate SL/2005/K/Sd5	-	EF174423	-	A,B	*Bos taurus*	-	-	-
Setaria equina (Abildgaard, 1789)	-	-	AJ544873	AJ544835	A,B,C	*Equus caballus*	-	-	-
Setaria labiatopapillosa (Alessandrini, 1848)	-	-	AJ544872	AJ544833	A,B,C	*Bos taurus*	-	-	-
Setaria tundra Issaitshikoff & Rajewskaya, 1928	SET1	MIB:Zpl:01159	AM749298	AM779848	A,B,C	*Capreolus capreolus*	2006	France	YES
Setaria tundra Issaitshikoff & Rajewskaya, 1928	-	-	AJ544874	AJ544834	A,B,C	*Capreolus capreolus*	-	-	-
Spirocerca lupi (Rudolphi, 1809)	Isolate 1	-	EF195132	-	A,B	*Canis lupus familiaris*	-	-	-
Spirocerca lupi (Rudolphi, 1809)	Isolate 2	-	EF394599	-	A,B	*Canis lupus familiaris*	-	-	-
Spirocerca lupi (Rudolphi, 1809)	Isolate 3	-	EF394600	-	A,B	*Canis lupus familiaris*	-	-	-
Spirocerca lupi (Rudolphi, 1809)	Isolate 4	-	EF394601	-	A,B	*Canis lupus familiaris*	-	-	-
Spirocerca lupi (Rudolphi, 1809)	Isolate 5	-	EF394602	-	A,B	*Canis lupus familiaris*	-	-	-
Spirocerca lupi (Rudolphi, 1809)	Isolate 6	-	EF394603	-	A,B	*Canis lupus familiaris*	-	-	-
Spirocerca lupi (Rudolphi, 1809)	Isolate 7	-	EF195133	-	A,B	*Canis lupus familiaris*	-	-	-
Spirocerca lupi (Rudolphi, 1809)	Isolate 8	-	EF394604	-	A,B	*Canis lupus familiaris*	-	-	-
Spirocerca lupi (Rudolphi, 1809)	Isolate 9	-	EF394605	-	A,B	*Canis lupus familiaris*	-	-	-
Spirocerca lupi (Rudolphi, 1809)	Isolate 10	-	EF394596	-	A,B	*Canis lupus familiaris*	-	-	-

Spirocerca lupi (Rudolphi, 1809)	Isolate 11	-	EF394597	-	A,B	*Canis lupus familiaris*	-	-	-
Spirocerca lupi (Rudolphi, 1809)	Isolate 12	-	EF394598	-	A,B	*Canis lupus familiaris*	-	-	-
Spirocerca lupi (Rudolphi, 1809)	Isolate 13	-	EF394606	-	A,B	*Canis lupus familiaris*	-	-	-
Spirocerca lupi (Rudolphi, 1809)	Isolate 14	-	EF394607	-	A,B	*Canis lupus familiaris*	-	-	-
Spirocerca lupi (Rudolphi, 1809)	Isolate 15	-	EF394608	-	A,B	*Canis lupus familiaris*	-	-	-
Spirocerca lupi (Rudolphi, 1809)	Isolate 16	-	EF394609	-	A,B	*Canis lupus familiaris*	-	-	-
Spirocerca lupi (Rudolphi, 1809)	Isolate 17	-	EF394610	-	A,B	*Canis lupus familiaris*	-	-	-
Spirocerca lupi (Rudolphi, 1809)	Isolate 18	-	EF394611	-	A,B	*Canis lupus familiaris*	-	-	-
Spirocerca lupi (Rudolphi, 1809)	Isolate 19	-	EF394612	-	A,B	*Canis lupus familiaris*	-	-	-
Spirocerca lupi (Rudolphi, 1809)	Isolate 20	-	EF394613	-	A,B	*Canis lupus familiaris*	-	-	-
Thelazia callipaeda Railliet & Henry, 1910	-	-	AJ544882	AJ544858	A,B,C	*Canis lupus familiaris*	-	-	-
Thelazia callipaeda Railliet & Henry, 1910	Haplotype h1	-	AM042549	-	A,B	n.d.	-	-	-
Thelazia callipaeda Railliet & Henry, 1910	Haplotype h2	-	AM042550	-	A,B	*Canis lupus familiaris*	-	-	-
Thelazia callipaeda Railliet & Henry, 1910	Haplotype h3	-	AM042551	-	A,B	*Canis lupus familiaris*	-	-	-
Thelazia callipaeda Railliet & Henry, 1910	Haplotype h4	-	AM042552	-	A,B	*Canis lupus familiaris*	-	-	-
Thelazia callipaeda Railliet & Henry, 1910	Haplotype h5	-	AM042553	-	A,B	*Canis lupus familiaris*	-	-	-
Thelazia callipaeda Railliet & Henry, 1910	Haplotype h6	-	AM042554	-	A,B	*Canis lupus familiaris*	-	-	-
Thelazia callipaeda Railliet & Henry, 1910	Haplotype h7	-	AM042555	-	A,B	*Canis lupus familiaris*	-	-	-
Thelazia callipaeda Railliet & Henry, 1910	Haplotype h8	-	AM042556	-	A,B	*Canis lupus familiaris*	-	-	-
Thelazia gulosa (Railliet & Henry, 1910)	-	-	AJ544881	AJ544857	A,B,C	*Bos taurus*	-	-	-
Thelazia lacrymalis (Gurlt, 1831)	-	-	AJ271619	AJ544856	A,B,C	*Equs caballus*	-	-	-
Wuchereria bancrofti (Cobbold, 1877)	-	-	AJ271612	AJ544844	A,B,C	*Homo sapiens*	-	-	-
Wuchereria bancrofti (Cobbold, 1877)	M005	MIB:Zpl:01169	AM749235	-	A,B	*Homo sapiens*	2000	Italy, Milan ****	YES
Wuchereria bancrofti (Cobbold, 1877)	M065	MIB:Zpl:01174	AM749235	-	A,B	*Homo sapiens*	2000	Italy, Milan ****	YES
