# Supplementary material for: Integrated taxonomy: traditional approach and DNA barcoding for the identification of filarioid worms and related parasites (Nematoda)
Source: Front Zool. 2009 Jan 7;6:1. doi: 10.1186/1742-9994-6-1 (PMC2657783; doi:10.1186/1742-9994-6-1)
Supplement: Additional File 2 — Experimental conditions. Detailed conditions of DNA extraction, primers, PCRs and DNA sequencing. [file 1742-9994-6-1-S2.pdf]

Detailed conditions of DNA extraction, primers, PCRs and DNA sequencing

For all the organisms examined, crude DNA preparations were obtained through proteinase-K treatment, according to [1].

coxI amplifications and sequences were obtained following [2]. In particular, the sequences have been generated using the primer pair: COIintF 5'-TGATTGGTGGTTTTGGTAA-3' and COIintR 5'-ATAAGTACGAGTATCAATATC-3'.

PCRs were performed in 20 µl volumes under the following final conditions: 1x buffer including 1.5 mM $MgCl_2$ (Master Taq kit, Eppendorf™), 0.2 mM of each dNTP, 1 µM each of forward and reverse primers, and 1 unit of polymerase (Master Taq kit, Eppendorf™). The thermal profile we used was: 94 °C 45 sec, 52 °C 45 sec, and 72 °C 90 sec for 40 cycles. The sequences obtained are about 650 bp long.

12S rDNA amplifications and sequences were obtained following [3]. In particular, the sequences have been generated using the primer pair 12SF: 5'-GTTCCAGAATAATCGGCTA-3' and 12SR: 5'-ATTGACGGATG(AG)TTTGTACC-3'.

PCRs were performed in 20 µl volumes under the following conditions: 1x buffer, 1.5 mM $MgCl_2$ (Master Taq kit, Eppendorf™), 0.2 mM of each dNTP, 1 µM of each primer, and 1 U of polymerase (Master Taq kit, Eppendorf™). The thermal profile was the following: 94°C 45 sec, 50°C 45 sec, and 72°C 90 sec for 40 cycles. The sequences obtained are about 450 bp long.

PCRs products were gel purified (using the Perfectprep Gel Cleanup, Eppendorf™) and directly sequenced with PCR primers in both directions using ABI technology.

REFERENCES

1. Bandi, C., Damiani, G., Magrassi, L., Gigolo, A., Fani R., Sacchi, L. **Flavobacteria as intracellular symbionts in cockroaches**. *Proc. R. Soc. Lond. B,* 1994. 257, 43-48.

2. Casiraghi, M., Anderson, T.J.C., Bandi, C., Bazzocchi, C., Genchi, C. **A phylogenetic analysis of filarial nematodes: comparison with the phylogeny of *Wolbachia* endosymbionts.** *Parasitol.* 2001. 122, 93-103.

3. Casiraghi M., Bain O., Guerrero R., Martin C., Pocacqua V., Gardner S.L., Franceschi A., Bandi C. **Mapping the presence of *Wolbachia pipientis* on the phylogeny of filarial nematodes: evidence for symbiont loss during evolution**. *Int J Parasitol.* 2004. 34: 191-203.
