# Supplementary figures and images for: Integrated taxonomy: traditional approach and DNA barcoding for the identification of filarioid worms and related parasites (Nematoda)
Source: Front Zool. 2009 Jan 7;6:1. doi: 10.1186/1742-9994-6-1 (PMC2657783; doi:10.1186/1742-9994-6-1)

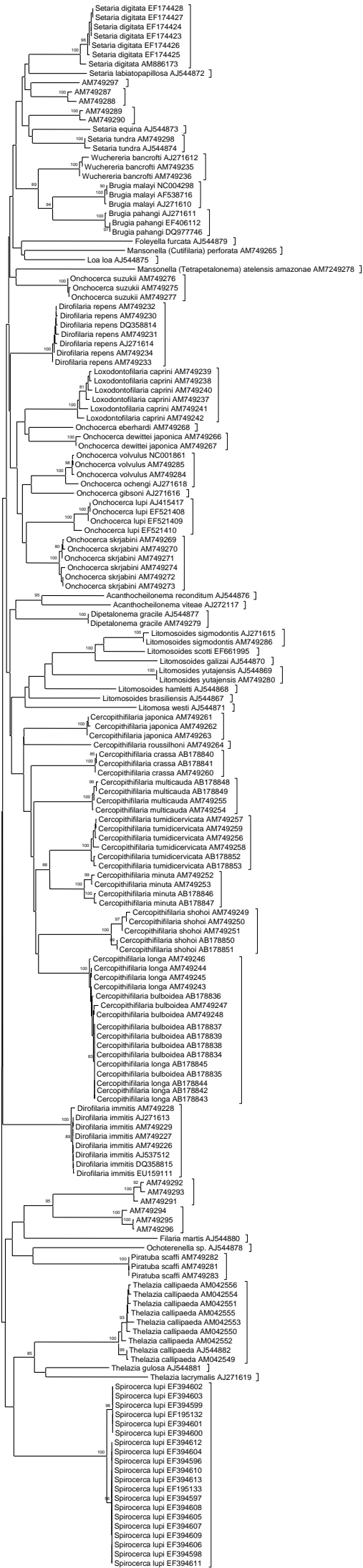

0.02

Supplement: Additional File 3 — NJ Tree. Neighbour joining tree based on coxI sequences generated using MEGA 4.0 (Tamura et al, 2007) – gaps treated as 'complete deletion'. [file 1742-9994-6-1-S3.pdf]
